# Functional Network Endophenotypes Unravel the Effects of Apolipoprotein E Epsilon 4 in Middle-Aged Adults

**DOI:** 10.1371/journal.pone.0055902

**Published:** 2013-02-12

**Authors:** Joseph S. Goveas, Chunming Xie, Gang Chen, Wenjun Li, B. Douglas Ward, Malgorzata B. Franczak, Jennifer L. Jones, Piero G. Antuono, Shi-Jiang Li

**Affiliations:** 1 Department of Psychiatry and Behavioral Medicine, Medical College of Wisconsin, Milwaukee, Wisconsin, United States of America; 2 Department of Biophysics, Medical College of Wisconsin, Milwaukee, Wisconsin, United States of America; 3 Department of Neurology, Medical College of Wisconsin, Milwaukee, Wisconsin, United States of America; Beijing Normal University, China

## Abstract

Apolipoprotein E-ε4 (APOE-ε4) accentuates memory decline, structural volume loss and cerebral amyloid deposition in cognitively healthy adults. We investigated whether APOE-ε4 carriers will show disruptions in the intrinsic cognitive networks, including the default mode (DMN), executive control (ECN) and salience (SN) networks, relative to noncarriers in middle-aged healthy adults; and the extent to which episodic-memory performance is related to the altered functional connectivity (Fc) in these networks. Resting-state functional connectivity MRI (R-fMRI) was used to measure the differences in the DMN, ECN and SN Fc between 20 APOE-ε4 carriers and 26 noncarriers. Multiple linear regression analyses were performed to determine the relationship between episodic-memory performance and Fc differences in the three resting-state networks across all subjects. There were no significant differences in the demographic and neuropsychological characteristics and the gray-matter volumes in the carriers and noncarriers. While mostly diminished DMN and ECN functional connectivities were seen, enhanced connections to the DMN structures were found in the SN in ε4 carriers. Altered DMN and ECN were associated with episodic memory performance. Significant Fc differences in the brain networks implicated in cognition were seen in middle-aged individuals with a genetic risk for AD, in the absence of cognitive decline and gray-matter atrophy. Prospective studies are essential to elucidate the potential of R-fMRI technique as a biomarker for predicting conversion from normal to early AD in healthy APOE-ε4 carriers.

## Introduction

Imaging genetics, a rapidly growing field in neuroscience, utilizes an imaging endophenotype to study the genetic influences on human cognitive function and behavior [1]. Imaging endophenotypes are commonly used as biomarkers in Alzheimer’s disease (AD) research because they are powerful techniques that can indicate disease-specific changes long before clinical evidence of neurodegeneration. Apolipoprotein E-ε4(APOE-ε4), the major known genetic susceptibility factor for late-onset AD, is associated with an accelerated decline in episodic memory, a dramatic increase in the risk of AD and a gene dose-dependent decrease in the age of onset [2], [3]. In presymptomatic APOE-ε4 carriers, enhanced gray-matter atrophy, greater β-amyloid deposition and diminished cerebral glucose metabolism in the same brain regions as AD patients are seen, although these findings are not universal [3]–[8]. Functional magnetic resonance imaging (fMRI) studies in APOE-**ε**4 carriers have shown differential patterns of brain activations during various memory tasks in the regions affected by AD, relative to noncarriers [9]–[13]. However, using traditional task-driven fMRI methods, it is difficult to reveal disruptions in the large-scale distributed brain networks associated with specific cognitive functions in persons at risk for AD [14]. Resting-state functional connectivity MRI (R-fMRI) is a powerful, task-independent imaging modality that examines the functional connections between anatomically distinct brain regions within specific networks and investigates the neural substrates of the interindividual phenotypic variation in normal and different disease states [15]–[20]. R-fMRI shows great promise as an imaging biomarker in preclinical and clinical AD states [21]–[30].

While the intrinsic effects of the ε4 allele on the functional architecture of the brain has primarily been demonstrated in the default mode network (DMN) [22], [23], [25], [27] and in nonAD-related resting-state networks (RSNs) [29], there are no studies to date that have identified functional connectivity (Fc) alterations in the other major cognitive networks, in middle-aged, cognitively intact APOE-ε4 carriers. Three major cognitive brain networks are identified in the resting brain; these include: (1) the DMN, which is involved in the episodic and autobiographical memory, semantic processing, self-monitoring, and related social cognitive functions; (2) the executive control network (ECN), which is critical for the control of working memory, and for judgment and decision making in the context of goal-directed behavior; and (3) the salience network (SN), which is involved in the orientation of attention to the most relevant internal brain events and external stimuli [30]–[32].

It is critical to study these three core cognitive RSNs in-depth because while episodic memory is the first cognitive domain to be affected, subtle nonamnestic deficits in executive control and attention are also found during the preclinical stages of AD [33], [34]. Recent evidence has also shown significant Fc alterations that extend beyond the DMN to affect the ECN and SN, in individuals with amnestic mild cognitive impairment (aMCI) and mild to moderate AD [30]. Functional alterations in the DMN and SN connections were also recently demonstrated in elderly APOE-ε4 carriers [35]. Interestingly, dynamic interactions have been identified between the DMN, ECN and SN in normal adults [32]. We postulated that the RSN disruptions are not isolated to the DMN and will extend beyond to involve the remainder of this tightly interconnected neural circuitry, even in middle-aged cognitively healthy ε4 carriers. The RSN disruptions are present even before gray-matter volume loss and cognitive decline are seen in genetically at-risk middle-aged individuals. These alterations, if identified, may provide a neural signature in identifying the individuals who are at increased risk for progression to AD.

The primary aim of this study was to test the hypothesis that while the greatest functional disruptions will be found in the DMN, significant Fc alterations also will be identified in the ECN and to a lesser degree in the SN in the APOE-ε4 carriers, preceding cognitive decline and gray-matter volume loss, relative to noncarriers. We also hypothesize that the alterations in these RSNs in the at-risk group will be associated with episodic memory performance.

## Materials and Methods

### Participants

A total of 48 middle-aged, cognitively healthy (CN) subjects between the ages of 44 and 65 years were enrolled in this study through advertisements and mailings. Written informed consent was obtained from all subjects in accordance with the Medical College of Wisconsin Institutional Review Board. Two subjects were excluded due to excessive motion during the MRI scan; therefore, the final sample consisted of a total of 46 subjects, who were included in this data analysis.

Demographic, family and medical histories were obtained from all subjects. All subjects denied impairment in memory or other cognitive skills, or activities of daily living and instrumental activities of daily living. They had no history of neurological disease, seizures, head injury with loss of consciousness within the past five years, stroke or transient ischemic attack, drug or alcohol abuse within the past five years, or major psychiatric disorders, including schizophrenia and other psychotic disorders, and mood disorders.

### Neuropsychological Assessment

All subjects had a normal neurological examination and a Hachinski Ischemia Score <1. Subjects scored within normal range on a brief battery of neuropsychological tests and the Beck Depression Inventory. Normal cognitive function was documented by scoring no more than 1 SD below the mean on the Rey Auditory-Verbal Learning Test (RAVLT) delayed recall and total learning (sum of trials 1–5), Boston Naming Test, Trail Making Test A & B and Digit Span Test scores [36]–[38]. **Table 1** summarizes the cognitive results for both groups. An experienced neurologist examined each subject, and two neurologists with expertise in evaluating persons with and without dementia reviewed the medical, neurological, functional and neuropsychological data, and consensus diagnoses were reached.

**Table 1:** Demographics and neuropsychological characteristics.

	APOE ε4 carriers (n = 20)		APOE-ε4 noncarriers (n = 26)		*p* value
	Mean	SD	Mean	SD	
**Gender, female/male**	14/6		17/9		NS†
**Age, year**	52.4	5.6	54.5	5.8	0.21*
**Education, year**	15.0	2.4	15.6	2.5	0.86*
**RAVLT delayed recall**	12.1	2.2	13.1	1.9	0.10*
**Digit Span**	16.6	3.6	18.5	3.4	0.07*
**Trail-making test**					
**A**	24.2	8.3	23.5	8.7	0.81*
**B**	56.0	19.4	50.2	18.7	0.31*
**Beck Depression**	3.0	3.3	3.5	3.9	0.64*

Note: No differences were found in gender, age, education, RAVLT delayed recall, digit span, trail-making A and B, and Beck depression scores between the APOE-ε4 carriers and noncarriers. Abbreviation: M, mean; SD, Standard Deviation; RAVLT, Rey Auditory Verbal Learning Test; NS, no significance;

† *p* value was obtained by *χ* ^2^ two-tailed test;

* *p* values were obtained by an independent-sample two-tailed *t* test. Unless otherwise indicated, data are presented as mean ± SD.

### APOE Genotyping

All participants provided a sample of DNA for APOE genotyping. The samples were collected by buccal swab, and analyzed for the presence of the APOE-ε2, APOE-ε3 and APOE-ε4 alleles. The material obtained was pooled and DNA was extracted, using the Gentra Systems Autopure LS DNA processing system, in the Medical College of Wisconsin Clinical and Translational Science Institute’s (CTSI) Core Laboratory. APOE genotyping from the extracted DNA was performed by restriction isotyping at the Oregon Health and Science University’s CTSI Core Laboratory [39], [40].

### MRI Acquisition

All scans were obtained using a whole-body 3T Signa GE scanner (Waukesha, WI) with a standard quadrature transmit-receive head coil. During the resting-state acquisitions, no specific cognitive tasks were performed, and the study participants were instructed to close their eyes, relax and stay awake. Sagittal resting-state fMRI datasets of the whole brain were obtained in six minutes with a single-shot gradient echo-echo planar imaging pulse sequence. The fMRI imaging parameters were: TE = 25 ms, TR = 2000 ms, flip angle of 90°; number of slices = 36; slice thickness 4 mm, matrix size = 64×64, field of view = 24×24 cm, total volumes = 180. High-resolution 3D spoiled gradient-recalled echo axial images were acquired for anatomical reference. The parameters were: TE/TR/TI = 4/10/450 ms; flip angle of 12°; number of slices = 144; slice thickness = 1 mm; matrix size = 256×192, FOV = 240×240 mm.

### Functional Connectivity MRI Processing and Analysis

#### MRI preprocessing

R-fMRI data analysis was performed, using Analysis of Functional NeuroImages (AFNI) software (http://afni.nimh.nih.gov/afni) and MatLab programs (MathWorks, Natick, MA). The spikes in the raw resting-state functional imaging data were removed (3dDespike); motion correction was performed by volume registration on R-fMRI data (3dvolreg); and detrending was carried out to remove Legendre polynomials (3dDetrend). Further, cardiac aliasing (AFNI command 3dretroicor -card) and the respiratory-volume variations were minimized (AFNI command 3dretroicor -resp). The signals in white matter and cerebrospinal fluid were regressed out, using averaged signals from the white matter and the ventricles, and the six-motion parameter vectors were regressed out from each voxel time series (program 3dDeconvolve) [41]–[45]. In addition, global signals were regressed out from the whole brain [19]. Finally, a bandpass filter was applied to keep only low-frequency fluctuations within the frequency range of 0.015 Hz and 0.1 Hz.

#### Resting-State functional connectivity analysis

Three seed regions of interest (ROI) were selected, based on previous publications [31], [46], [47]: DMN, posterior cingulate cortex (PCC) (Talairach coordinates: −1, −50, 26); ECN, right dorsolateral prefrontal cortex (DLPFC) (Talairach coordinates: 44, 36, 20); and SN, right orbital anterior insula (AI) (Talairach coordinates: 38, 26, −10). Six-millimeter spherical seed regions were placed at the above coordinates.

The various processing steps of seed-based voxelwise connectivity analysis for individual subjects have been described previously [48]–[50]. Briefly, coregistration and time course extraction (seed region, 3dfractionize) and voxelwise cross-correlation between the seed ROIs and the whole brain (3dfim^+^) were performed. The Pearson correlation coefficients were subjected to a Fisher’s *z* transformation, which yielded variants of normal distribution (3dcalc) [51]. The voxelwise values were spatially transformed from original to Talairach space (adwarp) and smoothed with a 6-mm Gaussian kernel in order to obtain RSN maps for each individual subject.

### Voxel-based Morphometry Analysis

Optimized voxel-based morphometry (VBM) analysis was performed, using the VBM toolbox in SPM8 (http://www.fil.ion.ucl.ac.uk/spm/software/spm8). The individual T1-weighted images for all subjects were segmented, normalized, and then, smoothed at 6-mm full-width half maximum. The gray-matter (GM) volume (modulated images) corresponding to the study group-specific template was extracted to determine the GM volume for the individual subject, and then, was entered into a two-sample *t*-test for group comparison.

### Statistical Analysis

#### Demographics and clinical performance comparisons

A two-sample *t*-test was used to test the differences in age, education, and behavior performances in RAVLT delayed recall (RAVLT-DR), Digit Span Test, Trail Making A & B, and the Beck Depression Inventory scores between APOE-ε4 carriers and noncarriers. Chi-square two-tail test was used to compare the group differences in gender.

#### Group-Level analysis for the functional connectivity network

Each of the three individual functional networks was obtained by two steps. First, the seed-based individual connectivity maps from each subject were grouped together according to APOE 4 carriers and noncarriers, respectively. Second, the patterns of each functional network were determined with a random-effects one-sample *t* test to identify significant clusters according to an AlphaSim program based on the Monte Carlo simulation algorithm (α = 0.05 voxelwise *p*<0.01, cluster size >1048 mm^3^) (http://afni.nimh.nih.gov/pub/dist/doc/manual/AlphaSim.pdf).

**Figure 1:**
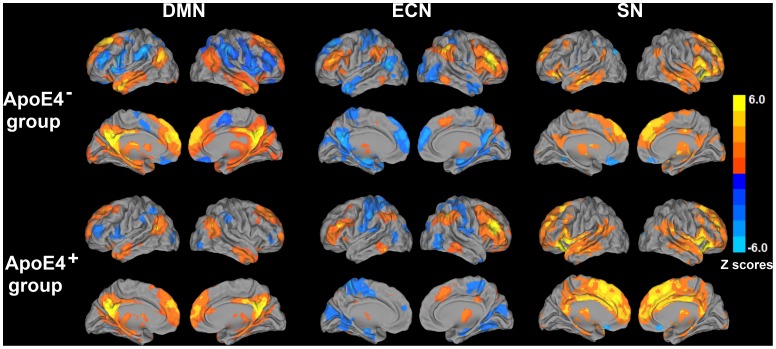
Functional connectivity pattern of the default mode network (DMN), executive control network (ECN), and salience network (SN) in APOE-ε4 carriers (ApoE4^+^) and nonAPOE-ε4 carriers (ApoE4^−^). Results are projected on a surface template (Caret software; Van Essen, 2005). Bright color indicates positive connectivity and the blue color indicates negative connectivity in ApoEε4^−^ and ApoEε4^+^ groups. Color bar is presented with *z* scores.

To find brain regions with significant group differences, the connectivity alteration for each network across all subjects was examined, using a two-sample *t* test (corrected with AlphaSim, α = 0.05, voxelwise *p*<0.05, cluster size >4048 mm^3^). For each subject, the average functional connectivity strengths were extracted from all regions that showed significant differences in the DMN, ECN and SN networks between carriers and noncarriers. In the DMN and ECN, regions that showed significant differences between groups were separated into positive and anticorrelated (or negative) networks, based on the functional connectivity patterns in the APOE-ε4 noncarriers (i.e., control) group (Figure 1). We repeated the two-sample *t* tests after controlling for age, gender, education and GM volumes.

#### Correlation of RAVLT-DR scores with the DMN, ECN, and SN network connectivity

To investigate the neural substrates underlying the functions of the RAVLT-DR scores on each network, a multiple linear regression analysis was employed (*3dRegAna*, AFNI) as follows:
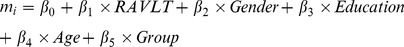



Where m_i_ is the m value of *i*th voxel across group subjects, β_0_ is the intercept of straight line fitting in the model. β_1_, β_2_, β_3_, β_4_ and β_5_ are the effects of RAVLT-DR scores, gender, education, age and individual groups on the functional connectivity strength of *i*th voxel within each network in the APOE-ε4 and noncarriers, respectively. The effects of β_2_, β_3_, β_4_ and β_5_ were discarded as covariance of no interest in the above linear regression model. The voxelwise multiple linear regression map was generated after multiple comparison correction (*AlphaSim,* AFNI, α = 0.05, voxelwise *p*<0.05 and cluster size >4,048 mm^3^) to identify the neural correlates of RAVLT-DR for each network **(Figure S1 for details on data analysis)**.

## Results

### Demographic and Neuropsychological Characteristics

Forty-six cognitively healthy adults were included in this data analysis. There were no statistical significant differences in age, gender, education, cognitive and depressive symptom scores (*p*>0.05) between groups, although APOE-ε4 carriers showed a trend toward lower RAVLT-DR (p = 0.10) and Digit Span Test (*p* = 0.07) scores, when compared to non ε4 carriers (**Table 1**).

### Neuroimaging Data

#### Gray-Matter volumes in APOE-ε4 carriers and noncarriers

Optimized VBM analysis showed no differences in GM volumes between groups (*p*>0.05; familywise error corrected). We also performed permutation tests for group comparison of GM volumes. Again, no significant differences were found between APOE4 carriers and noncarriers.

#### Resting-State networks in the APOE-ε4 carriers and noncarriers

(1) DMN: In the nonAPOE-ε4 carriers, positive correlations were seen between PCC and bilateral hippocampus/parahippocampal (Hip/PHG) region, superior frontal gyri (SFG), dorsomedial prefrontal cortex (DMPFC), middle temporal gyrus (MTG), caudate and thalamus. Anticorrelated networks were seen with bilateral insula, DLPFC, inferior parietal cortex (IPC) and ventromedial prefrontal cortex (VMPFC) (**Figure 1**). For the APOE-ε4 carriers, the DMN pattern also is shown in **Figure 1** **.**

**Figure 2:**
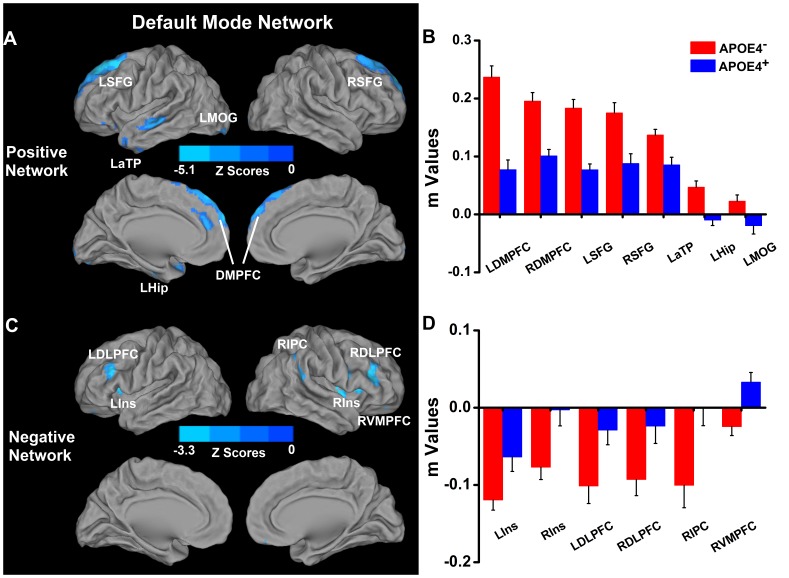
Differential connectivity of DMN between APOEε4^−^ and APOEε4^+^ groups. Left: location of significantly differential connectivity of DMN between ApoEε4^−^ and ApoEε4^+^ groups. Positive and negative networks were defined, based on the DMN pattern of ApoEε4^−^ subjects (please refer to Figure 1). Blue color indicates significantly decreased positive and anticorrelated (or negative) network connectivity of the DMN in the ApoEε4^+^ subjects in comparison to the ApoEε4^−^ subjects (**Figure 2A and 2C**). Right: Quantitative representation of differential connectivity of DMN between ApoEε4^−^ and ApoEε4^+^ groups (**Figure 2B and 2D**). Abbreviations: LSFG, left superior frontal gyrus; RSFG, right superior frontal gyrus; LMOG, left middle occipital gyrus; LaTP, left anterior temporal pole; LHip, left hippocampus; LDMPFC, left dorsomedial prefrontal cortex; RDMPFC, right dorsomedial prefrontal cortex; LDLPFC, left dorsolateral prefrontal cortex; RDLPFC, right dorsolateral prefrontal cortex; RIPC, right inferior parietal cortex; LIns, left insula; RIns, right insula; RVMPFC, right ventromedial prefrontal cortex. NOTE: increased connectivity in the bilateral caudate and left lentiform nucleus is not shown in the figure (see **Table S1**).

When compared with noncarriers, the high-risk group showed significantly decreased positive DMN connectivity in the bilateral DMPFC and SFG, and the left Hip/PHG, anterior temporal pole (aTP) and middle occipital gyrus (MOG), whereas increased positive connectivity was found in the left lentiform nucleus and bilateral caudate **(** **Figure 2A and 2B** **, Table S1)**. Also, diminished anticorrelated DMN connectivity was seen in insula and DLPFC bilaterally, and right IPC and VMPFC **(** **Figure 2C and 2D** **, Table S1**).

(2) ECN: The low-risk group showed positive correlations between right DLPFC and bilateral IPC, dorsal anterior cingulate cortex (dACC) and left DLPFC; and negative correlations between right DLPFC and bilateral hippocampus/parahippocampus, PCC, aTP, MTG, MOG, DMPFC and pre- and postcentral gyri **(** **Figure 1** **)**. For APOE-ε4 carriers, the ECN pattern is demonstrated in **Figure 1** **.**

**Figure 3:**
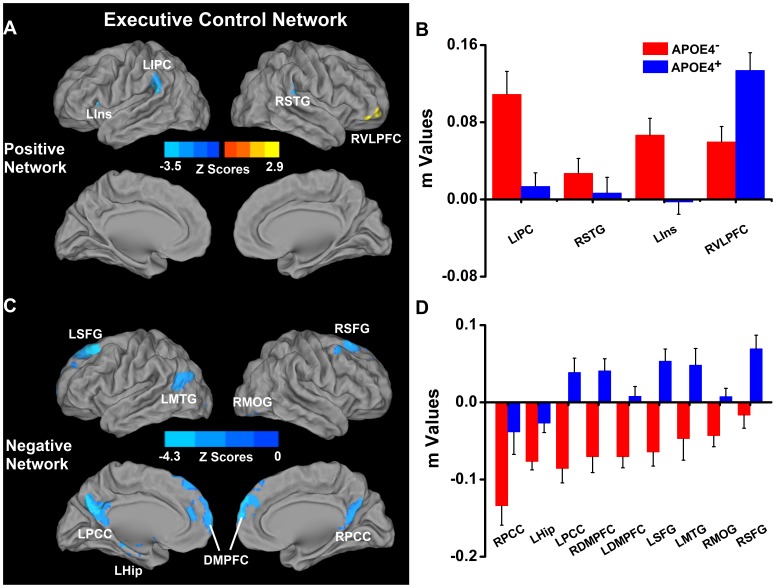
Differential connectivity of ECN between APOEε4^−^ and APOEε4^+^ groups. Left: location of significantly differential connectivity of ECN between ApoEε4^−^ and ApoEε4^+^ groups. Positive and negative networks were defined, based on the ECN pattern of the ApoEε4^−^ subjects (Figure 1). Blue color indicates significantly decreased connectivity of the positive and anticorrelation (or negative) networks of the ECN in the ApoEε4^+^ in comparison to the ApoEε4^−^ subjects (**Figure 3A and 3C**). Right: Quantitative representation of the differential connectivity of the ECN between ApoEε4^−^ and ApoEε4^+^ groups (**Figure 3B and 3D**). Abbreviations: LIPC, left inferior parietal cortex; RSTG, right superior temporal gyrus; LIns, left insula; RVLPFC, right ventrolateral prefrontal cortex; LSFG, left superior frontal gyrus; RSFG, right superior frontal gyrus; LMTG, left middle temporal gyrus; RMOG, right middle occipital gyrus; LPCC, left posterior cingulate cortex; RPCC, right posterior cingulate cortex; LHip, left hippocampus; LDMPFC, left dorsomedial prefrontal cortex; RDMPFC, right dorsomedial prefrontal cortex.

When compared with the control (nonε4 carrier group), the APOE-ε4 carriers showed decreased positive ECN connectivity in the left IPC and insula, and the right superior temporal gyrus (STG) (**Figure 3A and 3B** **, Table S2**). Also, *decreased* anticorrelated ECN connectivity was found in the bilateral SFG, DMPFC, PCC/precuneus and the MTG and hippocampus on the left, and the right MOG. Increased positive connectivity only was observed in the right ventrolateral prefrontal cortex (VLPFC) **(** **Figure 3C and 3D** **, Table S2)**.

(3) SN: In the control group, while the positively correlated network included bilateral DLPFC, IPC, DMPFC, PCC, thalamus, caudate and the middle cingulate cortex (MCC), the anticorrelated network included precuneus, parahippocampus and the insula on the left, and bilateral orbitofrontal cortex (OFC) **(** **Figure 1** **)**. For the APOE-ε4 carriers, the SN pattern is illustrated in **Figure 1** **.**

**Figure 4:**
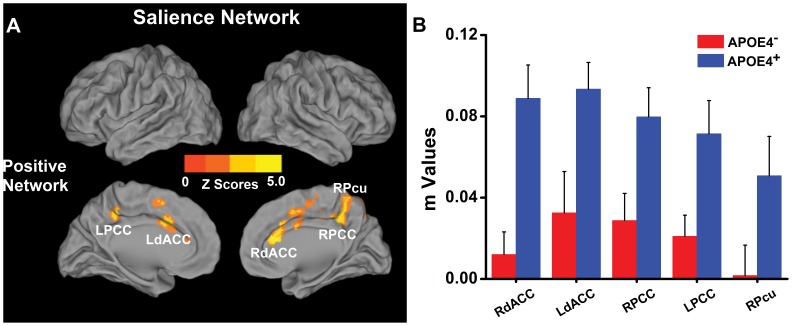
Differential connectivity of SN between APOEε4^−^ and APOEε4^+^ groups. Left: location of differential connectivity of DMN between ApoEε4^−^ and ApoEε4^+^ groups. Positive and negative networks were defined, based on the DMN pattern of the ApoEε4^−^ subjects (Figure 1). Bright color indicates significantly increased positive SN connectivity in the ApoEε4^+^ in comparison to the ApoEε4^−^ subjects (**Figure 4A**). No significant anticorrelation network differences of the SN were found between the groups. Right: Quantitative representation of differential SN connectivity between ApoEε4^−^ and ApoEε4^+^ groups (**Figure 4B**). Abbreviations: RdACC, right dorsal anterior cingulate cortex; LdACC, left dorsal anterior cingulate cortex; LPCC, left posterior cingulate cortex; RPCC, right posterior cingulate cortex; RPcu, right precuneus.

The APOE-ε4 carriers showed *increased* positively correlated network in the bilateral dACC, PCC and right precuneus, relative to the low-risk group **(** **Figure 4A and 4B** **, Table S3)**. There were no regions with diminished AI connectivity, and no differences in the anticorrelated networks were observed.

All the above findings remained significant, even after controlling for age, gender, education and GM volumes.

Since the effects of head motion on the intrinsic functional connectivity is a concern [52], [53], we performed the group-level analyses between APOE-**ε**4+ and APOE-**ε**4- on the DMN, ECN and SN after controlling the effects of head motion, using the root mean square as no interest covariates, respectively. We did not find significant changes of group-level comparison on the DMN, ECN, and SN with or without regressing out motion effects in our dataset.

#### Exploratory analysis of the effects of RAVLT-DR scores on the RSNs across all subjects

**Figure 5:**
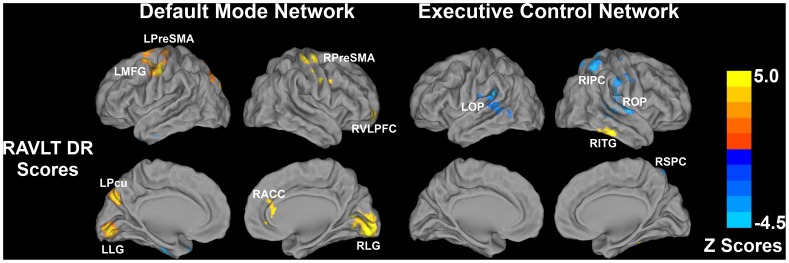
Behavioral significance of the intrinsic DMN and ECN connectivity across all subjects. Significant correlation between RAVLT delayed recall scores and the intrinsic connectivity of DMN and ECN was evident. No significant correlation was found between RAVLT delayed recall scores and the intrinsic connectivity of the SN. Bright color indicates positive correlation and blue color indicates negative correlation across all subjects. Color bar is presented with *z* scores. Abbreviation: RAVLT DR, Rey Auditory-Verbal Learning Test delayed recall; LPreSMA, left presomatomotor area; LMFG, left middle frontal gyrus; LPcu, left precuneus; LLG, left lingual gyrus; RPreSMA, right presomatomotor area; RVLPFC, right ventrolateral prefrontal cortex; RACC, right anterior cingulate cortex; RLG, right lingual gyrus; LOP, left operculum; ROP, right operculum; RIPC, right inferior parietal cortex; RITG, right inferior temporal gyrus; RSPC, right superior parietal cortex.

Episodic memory function is the earliest cognitive measure that shows decline in genetically predisposed individuals at risk for AD. Therefore, we did an exploratory analysis to identify the extent to which this cognitive domain is related to the altered Fc in the three networks across all subjects, after controlling for age, gender, education and subject group effects **(** **Figure 5** **and Table S4)**.

1. DMN: RAVLT-DR scores were positively correlated to the presomatomotor area (PreSMA) and lingual gyrus (LG) bilaterally; MFG and precuneus on the left; and ventrolateral PFC (VLPFC) and ACC on the right.
2. ECN: While the RAVLT-DR scores were negatively correlated to the operculum clusters bilaterally and right IPC/superior parietal cortex (SPC) cluster, memory performance was positively correlated to the right inferior temporal gyrus (ITG). We did not find any relationship between RAVLT-DR scores and SN Fc in any regions.

## Discussion

This is the first study to comprehensively identify functional connectivity alterations in the three RSNs implicated in cognition, in middle-aged adults with genetic risk for AD, preceding cognitive decline and gray-matter atrophy. We found the greatest Fc disruptions in the DMN, followed by the ECN and the least in the SN. We also have preliminary evidence to show that the alterations in the DMN and ECN are associated with memory performance in certain frontal, parietal and temporal regions across all subjects. The R-fMRI technique has great potential as an imaging endophenotype in preclinical AD research.

Our findings of diminished correlated and anticorrelated DMN Fc in APOE-ε4 carriers, especially in the frontal lobe and medial temporal lobe (MTL) structures, have been previously demonstrated in the absence of brain fibrillar β-amyloid plaque deposition [25]. Also, diminished DMN Fc was also found in elderly APOE-ε4 carriers [35]. In contrast, while predominantly increased DMN connections to the prefrontal cortices and MTL regions were seen in young healthy **ε**4 carriers [22], APOE status had no effect on the DMN in another study [29]. The inconsistencies may be a result of the differences in the age of enrolled participants [22] and the methodology used to identify the RSNs (i.e., the independent component analysis [29] versus the seed-based ROI analysis used in our study). Regardless, we found increases in PCC Fc, though this was limited to the basal ganglia structures. We believe that these increased functional connections in the DMN reflect the compensatory recruitment of additional neural resources by which the high-risk subjects retain normal cognitive functions, as previously described in fMRI task-activation and resting-state studies [9], [10], [12], [23], [30].

We demonstrate for the first time that the core cognitive RSN disruptions extend beyond the DMN to include the ECN and SN in middle-aged individuals at genetic risk for AD. Structures within the task-positive ECN were significantly correlated to each other; the ECN seed was anticorrelated with brain regions in the DMN in our control group. This is consistent with the literature [18]. We believe that the intactness of the correlated and anticorrelated functional connectivity patterns in these RSNs is critical for performing episodic and working memory, and executive control functions in healthy individuals. Interestingly, while we did not observe significant differences in the cognitive measures between the APOE-ε4 carriers and noncarriers, decreases in the ECN connectivity were seen in the high-risk group. In AD, increased ECN connectivity previously has been reported relative to cognitively healthy normal persons [30], [54], [55]. Moreover, the increased frontal network connectivity observed in AD is more extensive, in comparison to aMCI and controls [30]. Patients with mild AD rely on increased ECN connectivity to compensate for a poorly functioning network (e.g., the DMN) as an attempt to improve cognitive performance. Our findings suggest that the increases in the ECN connections in the genetically at-risk individuals only may occur when cognitive decline is imminent and in those who have incipient AD. Future longitudinal studies are essential to elucidate this hypothesis.

The increased connections between the right AI with the dorsal ACC, PCC and precuneus, show for the first time that close functional interactions exist between the DMN and the SN in middle-aged individuals at high risk for AD. AI and ACC, core regions of the SN, are often coactivated during various internal and external salient stimuli. They play an important role in guiding behaviors associated with social, affective and higher order mental processes. AI, a crucial hub of the SN, is thought to mediate the dynamic interactions between other networks implicated in cognition, mainly the DMN and ECN. Increased Fc in the SN and decreased DMN Fc have been previously described in elderly cognitively healthy APOE-ε4 carriers and in patients with mild AD [35], [55]. More sophisticated Granger causality analyses are needed to further address the dynamic interactions between these RSNs in individuals at risk for AD [56]. It is significant that the ECN and SN are originally defined with the right DLPFC and right AI as the seed regions according to the task-driven fMRI studies [31], [57]–[59]. In using the left DLPFC and left AI as seeds, different results were found (see **Table S5**, **Figure S2** and **Figure S3**). Further study is needed to address neurobiological significance.

The mechanisms that contribute to the Fc differences in the cognition-related neuronal circuits between carriers and noncarriers are not fully understood. One explanation is that the APOE-**ε**4 isoform increases β-amyloid aggregation in brain regions involved in these RSNs, thus leading to the observed functional disconnections. Aβ plaque deposition can precede neurodegeneration and clinical symptom onset in middle-aged adults at risk for AD. Interestingly, the MTL and neocortical areas that are functionally altered in the three RSNs also are early targets of β-amyloid plaque deposition [60]–[62]. In contrast, occipital lobe structures are only affected in the advanced phases of AD [63]. Moreover, alterations in noncognitive-associated RSNs are found in healthy **ε**4-carriers [29], and diminished DMN Fc is seen in the absence of cerebral amyloid pathology [25]. These findings suggest that the effects of APOE genetic status on the functional connections may not be fully mediated by amyloid pathology.

APOE also plays a critical role in the neuronal development, and the presence of **ε**4-allele is found to decrease neuronal growth and synaptic plasticity [64], [65], thereby detrimentally affecting functional integration in cognitive networks by nonβ-amyloid-dependent mechanisms. If so, it is plausible that the loss of functional integration, as evidenced by R-fMRI alterations in **ε**4-carriers, may precede commonly used AD biomarkers, including cerebral β-amyloid deposition, structural atrophy and neuropsychological deficits. APOE-**ε**4 also stimulates abnormal tau hyperphosphorylation and formation of neurofibrillary tangles (NFTs) [66]. Finally, APOE-**ε**4 impairs cholesterol delivery from astrocytes to neurons, and can accentuate neurodegeneration [67], [68].

Our exploratory voxel-based whole-brain analyses showed a weak but statistically significant relationship between episodic memory performance and the DMN and ECN Fc in several cortical areas across the whole sample. Interestingly, the brain areas within these cognitive circuits that are recruited to perform episodic memory functions across all participants, after controlling for several factors, including the group status, do not overlap with the areas differentially affected in carriers. These results indicate that distinct brain structures are often involved in performing episodic memory-related functions in cognitively healthy normal individuals and APOE-**ε**4 carriers, further providing evidence of the brain’s ability to balance and reorganize in normal and at-risk disease states.

Our study is not without limitations. First, this is a cross-sectional study. Future longitudinal studies are needed to disentangle the mechanisms linking APOE status, baseline cognition and brain function with the future incidence of AD. Second, while the lack of knowledge regarding the cerebral amyloid burden status is a limitation, DMN connectivity differences observed in this study are consistent with the findings reported in a sample free of cortical β-amyloid [25]. Third, in the ROI-based DMN analysis, different seeds have been used to study Fc, and it is possible that distinct locations may produce divergent results. However, our findings are consistent with recent observations that have used different DMN and SN seed regions [25], [35]. Because our analysis is hypothesis driven, we limited our analysis to three RSNs; we plan to perform a data-driven, large-scale functional connectivity analysis in this cohort [69]. Fourth, cerebrovascular risk factors and disease are related to memory decline in **ε**4-allelic carriers [70], [71]. Although our study did not enroll individuals with significant vascular disease, future studies need to examine the relationship of varying degrees of white-matter disease and altered structural connectivity with Fc differences in cognitively healthy **ε**4-allelic carriers. Fifth, because the primary objective of this study was to determine the effects of APOE on Fc in the cognitive RSNs, we did not examine the influence of family history of AD on the functional architecture of these networks. Finally, a gene dose-dependent relationship on RSN Fc may exist in APOE carriers [29]. We were unable to explore this further because of small samples in **ε**2/**ε**3 (N = 2) and **ε**4/**ε**4 (N = 4) groups.

### Conclusions

In summary, we have shown for the first time that intrinsic functional connectivity alterations is present in the core RSNs implicated in cognition beyond the DMN, even before gray-matter volume loss and the onset of cognitive decline, in middle-aged, **ε**4 carriers. Functional connectivity endophenotypes that are identified, using R-fMRI, can unravel the influence of APOE in middle-aged, cognitively healthy individuals with a genetic risk for AD. We also show the preliminary evidence of the effects of episodic memory performance on the altered Fc in the DMN and ECN, in cognitively normal individuals. Prospective studies that address the use of R-fMRI technique as a potential biomarker for predicting conversion from normal to early AD is needed, in **ε**4 carriers.

## Supporting Information

Figure S1 **Flowchart of data process.** Abbreviation: SPGR, spoiled gradient-recalled echo sequence; fMRI: functional magnetic resonance imaging; PCC, posterior cingulate cortex; AI, anterior insula; R DLPFC, right dorsolateral prefrontal cortex; DMN, default mode network; ECN, executive control network; SN, salience network; RAVLT-DR: Rey auditory verbal learning test delayed recall.
(DOC)

Figure S2 **Patterns of Left DLPFC and Left Anterior Insula Functional Networks in APOE-ε4 carriers (ApoE4+) and APOE-ε4 noncarriers (ApoE4-) (** ***p*** **<0.05, corrected with AlphaSim).** Results are projected on a surface template (Caret software; Van Essen, 2005). Bright color indicates positive connectivity and blue color indicates negative connectivity in ApoEε4^−^ and ApoEε4^+^ groups. Color bar is presented with *z* scores. Abbreviation: DLPFC, dorsolateral prefrontal cortex; AI, anterior insula.
(DOC)

Figure S3 **Functional Connectivity Differences of Left DLPFC Network and Left AI Network between APOE-ε4 carriers and noncarriers (** ***p*** **<0.05, corrected with AlphaSim).** In the left DLPFC network, the APOE-ε4 carriers showed significantly diminished connectivity in the bilateral superior prefrontal cortex (LSFG/RSFG) and anterior temporal pole (LaTP/RaTP), and right posterior middle temporal gyrus (RpMTG). In the left AI network, significantly decreased functional connections were found in the bilateral PCC (LPCC/RPCC), right DLPFC (RDLPFC) and cuneus (RCun), left inferior parietal cortex/posterior middle temporal gyrus (LIPC/pMTG), and left ventral medial prefrontal cortex (vmPFC), relative to noncarriers. Blue color indicates decreased connectivity of left DLPFC network and left AI network in ApoEε4 carriers compared to ApoEε4 non-carriers. Color bar is presented with *z* scores. Abbreviation: DLPFC, dorsolateral prefrontal cortex; AI, anterior insula.
(DOC)

Table S1 **Differential connectivity of DMN in APOEε4 carriers compared with non ε4 carriers.** Notes: x,y,z, coordinates of primary peak locations in the Talairach space. Abbreviation: BA, Brodmann area; L/R, left/Right; DMPFC, dorsomedial prefrontal cortex; SFG, superior frontal gyrus; MOG, middle occipital gyrus; Hip/PHG, hippocampus/parahippocampal gyrus; aTP, anterior temporal pole; DLPFC, dorsolateral prefrontal cortex; IPC, inferior parietal cortex; VMPFC, ventromedial prefrontal cortex.
(DOC)

Table S2 **Differential connectivity of ECN in APOEε4 carriers compared with non-ε4 carriers.** Notes: x,y,z, coordinates of primary peak locations in the Talairach space. Abbreviation: BA, Brodmann area; L/R, left/right; IPC, inferior parietal cortex; STG, superior temporal gyrus; VLPFC, ventrolateral prefontal cortex; SFG, superior frontal gyrus; DMPFC, dorsomedial prefrontal cortex; MOG, middle occipital gyrus; PCC, posterior cingulate cortex; Hip/PHG, hippocampus/parahippocampal gyrus; MTG, middle temporal gyrus.
(DOC)

Table S3 **Differential connectivity of SN in APOEε4 carriers compared with non-ε4 carriers.** Notes: x,y,z, coordinates of primary peak locations in the Talairach space. Abbreviation: BA, Brodmann area; L/R, left/right; dACC, dorsal anterior cingulate cortex; PCC, posterior cingulate cortex.
(DOC)

Table S4 **The effects of RAVLT scores on the DMN and ECN across all subjects.** Notes: x,y,z, coordinates of primary peak locations in the Talairach space. Abbreviation: BA, Brodmann area; L/R, left/right; MFG, middle frontal gyrus; PreSMA, pre-somatomotor area; PCC, posterior cingulate cortex; VLPFC, ventrolateral prefrontal cortex; ACC, anterior cingulate cortex; IPC, inferior parietal cortex; SPC, superior parietal cortex; ITG, inferior temporal gyrus.
(DOC)

Table S5 **Differential connectivity of left DLPFC network and left AI network in APOEε4 carriers compared with non-ε4 carriers.** Notes: x,y,z, coordinates of primary peak locations in the Talairach space. Abbreviation: DLPFC, dorsolateral prefrontal cortex; AI, anterior insula; BA, Brodmann area; L/R, left/right; SFG, superior frontal gyrus; aTP, anterior temporal pole; pMTG, posterior middle temporal gyrus; PCC, posterior cingulate cortex; vmPFC, ventromedial prefrontal cortex; IPC, inferior parietal cortex; AG, angular gyrus.
(DOC)
