# Supplementary figures and images for: Functional Network Endophenotypes Unravel the Effects of Apolipoprotein E Epsilon 4 in Middle-Aged Adults
Source: PLoS One. 2013 Feb 12;8(2):e55902. doi: 10.1371/journal.pone.0055902 (PMC3570545; doi:10.1371/journal.pone.0055902)

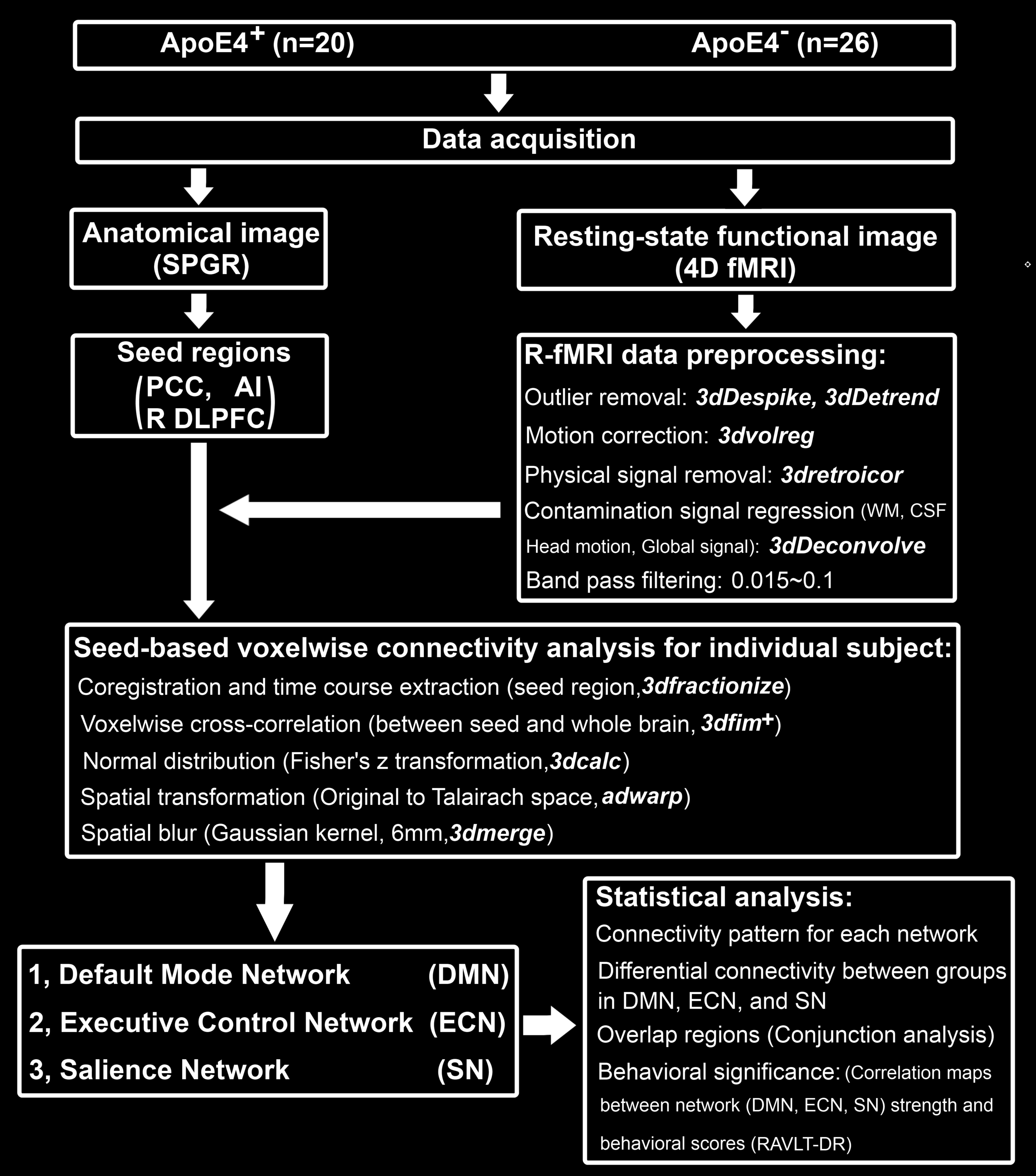


**Figure S1.**

Supplement: Figure S1 — Flowchart of data process. Abbreviation: SPGR, spoiled gradient-recalled echo sequence; fMRI: functional magnetic resonance imaging; PCC, posterior cingulate cortex; AI, anterior insula; R DLPFC, right dorsolateral prefrontal cortex; DMN, default mode network; ECN, executive control network; SN, salience network; RAVLT-DR: Rey auditory verbal learning test delayed recall. (DOC) [file pone.0055902.s001.doc]

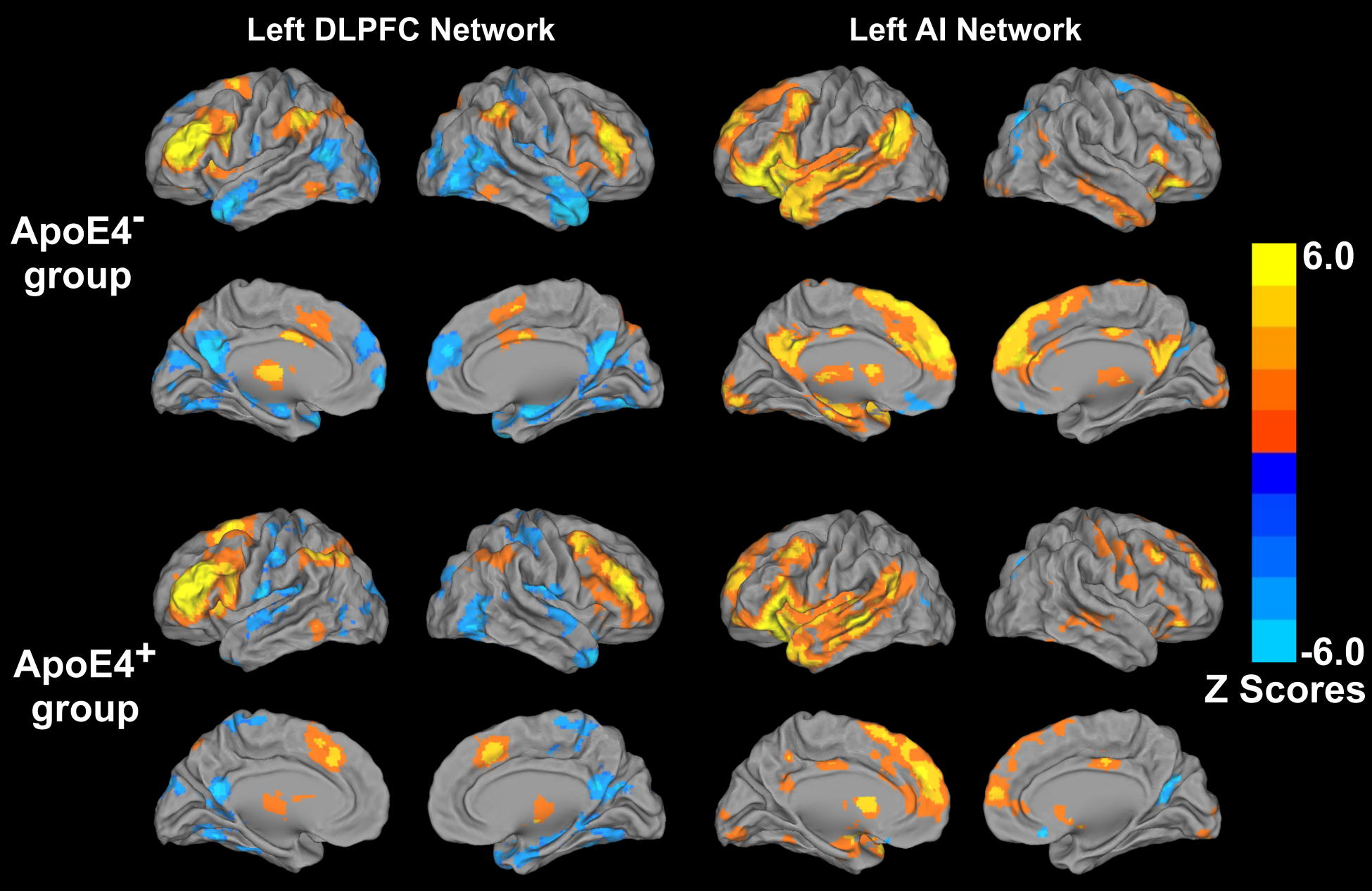


Figure S2.

Supplement: Figure S2 — Patterns of Left DLPFC and Left Anterior Insula Functional Networks in APOE-ε4 carriers (ApoE4+) and APOE-ε4 noncarriers (ApoE4-) ( p <0.05, corrected with AlphaSim). Results are projected on a surface template (Caret software; Van Essen, 2005). Bright color indicates positive connectivity and blue color indicates negative connectivity in ApoEε4− and ApoEε4+ groups. Color bar is presented with z scores. Abbreviation: DLPFC, dorsolateral prefrontal cortex; AI, anterior insula. (DOC) [file pone.0055902.s002.doc]

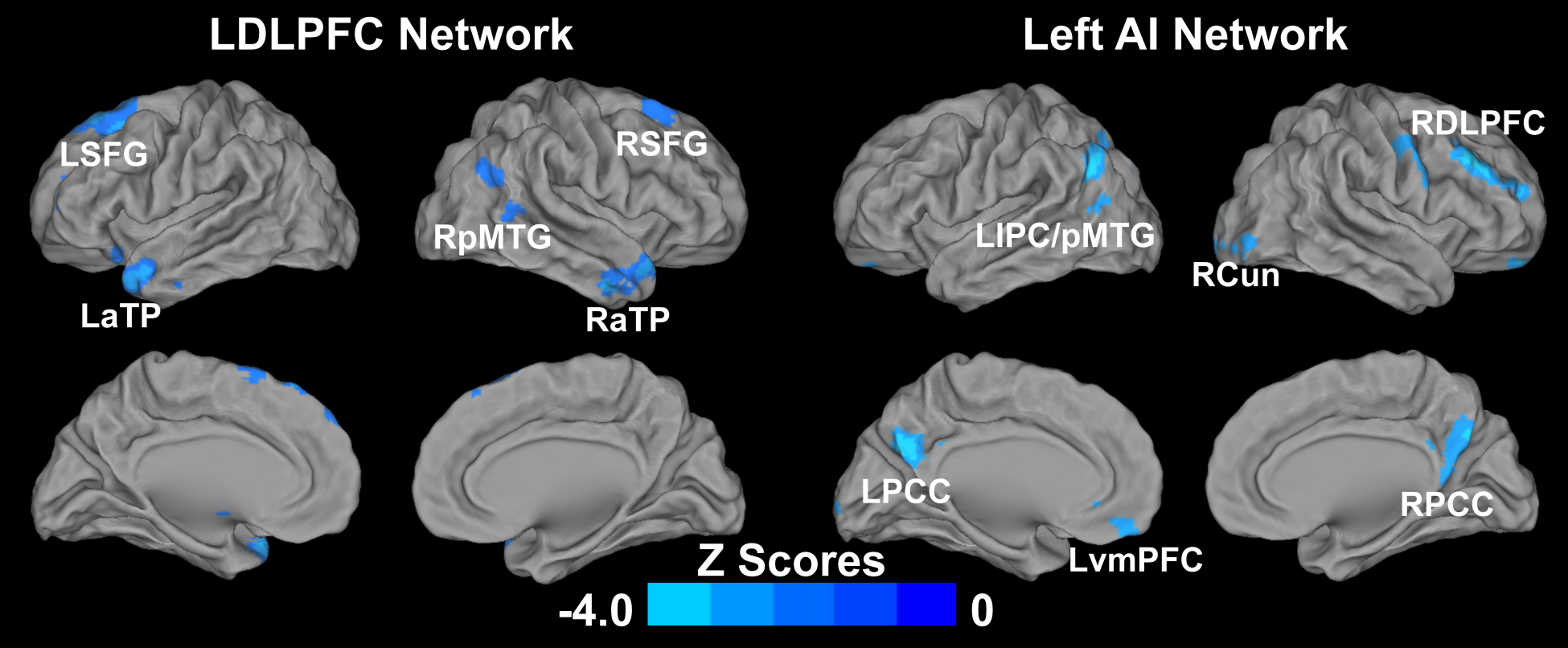


Figure S3.

Supplement: Figure S3 — Functional Connectivity Differences of Left DLPFC Network and Left AI Network between APOE-ε4 carriers and noncarriers ( p <0.05, corrected with AlphaSim). In the left DLPFC network, the APOE-ε4 carriers showed significantly diminished connectivity in the bilateral superior prefrontal cortex (LSFG/RSFG) and anterior temporal pole (LaTP/RaTP), and right posterior middle temporal gyrus (RpMTG). In the left AI network, significantly decreased functional connections were found in the bilateral PCC (LPCC/RPCC), right DLPFC (RDLPFC) and cuneus (RCun), left inferior parietal cortex/posterior middle temporal gyrus (LIPC/pMTG), and left ventral medial prefrontal cortex (vmPFC), relative to noncarriers. Blue color indicates decreased connectivity of left DLPFC network and left AI network in ApoEε4 carriers compared to ApoEε4 non-carriers. Color bar is presented with z scores. Abbreviation: DLPFC, dorsolateral prefrontal cortex; AI, anterior insula. (DOC) [file pone.0055902.s003.doc]
