# Supplementary material for: Functional Network Endophenotypes Unravel the Effects of Apolipoprotein E Epsilon 4 in Middle-Aged Adults
Source: PLoS One. 2013 Feb 12;8(2):e55902. doi: 10.1371/journal.pone.0055902 (PMC3570545; doi:10.1371/journal.pone.0055902)
Supplement: Table S1 — Differential connectivity of DMN in APOEε4 carriers compared with non ε4 carriers. Notes: x,y,z, coordinates of primary peak locations in the Talairach space. Abbreviation: BA, Brodmann area; L/R, left/Right; DMPFC, dorsomedial prefrontal cortex; SFG, superior frontal gyrus; MOG, middle occipital gyrus; Hip/PHG, hippocampus/parahippocampal gyrus; aTP, anterior temporal pole; DLPFC, dorsolateral prefrontal cortex; IPC, inferior parietal cortex; VMPFC, ventromedial prefrontal cortex. (DOC) [file pone.0055902.s004.doc]

**Table S1.**

| Brain region | Side | BA | Cluster  Size  (mm^3^) | Talairach coordinates  (LPI) | | | Z Score |
| --- | --- | --- | --- | --- | --- | --- | --- |
|  |  |  |  | x | y | z |  |
| **Decreased Positive Network** | | | | | | | |
| DMPFC | L/R | 8 | 35976 | -11 | 43 | 42 | -4.52 |
| SFG | L/R | 8 |  | -21 | 26 | 49 | -4.11 |
| MOG | L | 18 | 17632 | 49 | -75 | -9 | -2.96 |
| Hip/PHG | L |  | 9680 | -33 | -13 | -22 | -3.79 |
| aTP | L | 22/38 |  | -45 | 15 | -28 | -3.08 |
| **Decreased Anticorrelation Network** | | | | | | | |
| Insula | L/R | 13 | 8112 | 41 | 1 | 4 | -3.39 |
| DLPFC | L/R | 46 | 4592 | 42 | 33 | 18 | -3.06 |
| IPC | R | 40 | 5160 | 61 | -39 | 26 | -3.46 |
| VMPFC | R | 11 | 7376 | 31 | 38 | -13 | -3.89 |
| **Increased Positive Network** | | | | | | | |
| Lentiform Nucleus | L |  | 5280 | -27 | -13 | -4 | 3.30 |
| Caudate | L/R |  |  | -7 | 2 | 13 | 2.25 |
