# Supplementary material for: Functional Network Endophenotypes Unravel the Effects of Apolipoprotein E Epsilon 4 in Middle-Aged Adults
Source: PLoS One. 2013 Feb 12;8(2):e55902. doi: 10.1371/journal.pone.0055902 (PMC3570545; doi:10.1371/journal.pone.0055902)
Supplement: Table S2 — Differential connectivity of ECN in APOEε4 carriers compared with non-ε4 carriers. Notes: x,y,z, coordinates of primary peak locations in the Talairach space. Abbreviation: BA, Brodmann area; L/R, left/right; IPC, inferior parietal cortex; STG, superior temporal gyrus; VLPFC, ventrolateral prefontal cortex; SFG, superior frontal gyrus; DMPFC, dorsomedial prefrontal cortex; MOG, middle occipital gyrus; PCC, posterior cingulate cortex; Hip/PHG, hippocampus/parahippocampal gyrus; MTG, middle temporal gyrus. (DOC) [file pone.0055902.s005.doc]

**Table S2.**

| Brain region | Side | BA | Cluster  Size  (mm^3^) | Talairach coordinates  (LPI) | | | Z Score |
| --- | --- | --- | --- | --- | --- | --- | --- |
|  |  |  |  | x | y | z |  |
| **Decreased Positive Network** | | | | | | | |
| IPC | L | 42/40 | 9600 | -58 | -12 | 13 | -3.63 |
| Insula | L | 13 |  | -31 | 14 | 11 | -3.24 |
| STG | R | 13 | 5080 | 45 | -5 | 10 | -3.46 |
| **Increased Positive Network** | | | | | | |  |
| VLPFC | R | 10 |  | 31 | 53 | -3 | 3.29 |
| **Decreased Anticorrelation Network** | | | | | | | |
| SFG | L/R | 8 | 29896 | -25 | 27 | 48 | -4.85 |
| DMPFC | L/R | 10 |  | 5 | 63 | 22 | -3.83 |
| MOG | R | 18 | 17136 | 45 | -77 | -10 | -3.09 |
| PCC/Precuneus | L/R | 31/7 | 15922 | -9 | -63 | 30 | -3.91 |
| Hip/PHG | L | 28 |  | -21 | -23 | -10 | -3.31 |
| MTG | L | 39 | 7304 | -41 | -63 | 26 | -2.99 |
