# Supplementary material for: Functional Network Endophenotypes Unravel the Effects of Apolipoprotein E Epsilon 4 in Middle-Aged Adults
Source: PLoS One. 2013 Feb 12;8(2):e55902. doi: 10.1371/journal.pone.0055902 (PMC3570545; doi:10.1371/journal.pone.0055902)
Supplement: Table S3 — Differential connectivity of SN in APOEε4 carriers compared with non-ε4 carriers. Notes: x,y,z, coordinates of primary peak locations in the Talairach space. Abbreviation: BA, Brodmann area; L/R, left/right; dACC, dorsal anterior cingulate cortex; PCC, posterior cingulate cortex. (DOC) [file pone.0055902.s006.doc]

**Table S3.**

| Brain region | Side | BA | Cluster  Size  (mm^3^) | Talairach coordinates  (LPI) | | | Z Score |
| --- | --- | --- | --- | --- | --- | --- | --- |
|  |  |  |  | x | y | z |  |
| **Increased Positive Network** | | | | | | | |
| dACC | L/R | 24/32 | 7432 | 7 | 31 | 16 | 4.03 |
| PCC | L/R | 31 | 5560 | 7 | -37 | 39 | 3.02 |
| Precuneus | R | 7 |  | 9 | -71 | 42 | 2.63 |
