# Supplementary material for: Functional Network Endophenotypes Unravel the Effects of Apolipoprotein E Epsilon 4 in Middle-Aged Adults
Source: PLoS One. 2013 Feb 12;8(2):e55902. doi: 10.1371/journal.pone.0055902 (PMC3570545; doi:10.1371/journal.pone.0055902)
Supplement: Table S4 — The effects of RAVLT scores on the DMN and ECN across all subjects. Notes: x,y,z, coordinates of primary peak locations in the Talairach space. Abbreviation: BA, Brodmann area; L/R, left/right; MFG, middle frontal gyrus; PreSMA, pre-somatomotor area; PCC, posterior cingulate cortex; VLPFC, ventrolateral prefrontal cortex; ACC, anterior cingulate cortex; IPC, inferior parietal cortex; SPC, superior parietal cortex; ITG, inferior temporal gyrus. (DOC) [file pone.0055902.s007.doc]

**Table S4.**

| Brain region | Side | BA | Cluster  Size  (mm^3^) | Talairach coordinates | | | Z Score |
| --- | --- | --- | --- | --- | --- | --- | --- |
|  |  |  |  | x | y | z |  |
| **Default mode network** |  |  |  |  |  |  |  |
| MFG | L | 6 | 16088 | -30 | -6 | 57 | 3.56 |
| PreSMA | L/R | 6/4 |  | -33 | -9 | 56 | 3.38 |
| Lingual gyrus | L/R | 18 | 12912 | 3 | -75 | -8 | 3.93 |
| PCC/Precuneus | L | 31 | 6928 | -13 | -67 | 26 | 4.33 |
| VLPFC | R | 10 | 4264 | 25 | 53 | -2 | 3.38 |
| ACC | R | 32/24 |  | 7 | 35 | 19 | 2.39 |
| **Executive control network** | | | | | | | |
| Operculum | R | 13/22/21 | 12448 | 40 | -20 | 12 | -2.41 |
| Operculum | L | 13/22 | 8504 | -40 | -27 | 17 | -4.37 |
| IPC/SPC | R | 7/40 | 6384 | 30 | -47 | 51 | -3.24 |
| ITG | R | 20 | 5888 | 63 | -35 | -18 | 4.02 |
