# Supplementary material for: Functional Network Endophenotypes Unravel the Effects of Apolipoprotein E Epsilon 4 in Middle-Aged Adults
Source: PLoS One. 2013 Feb 12;8(2):e55902. doi: 10.1371/journal.pone.0055902 (PMC3570545; doi:10.1371/journal.pone.0055902)
Supplement: Table S5 — Differential connectivity of left DLPFC network and left AI network in APOEε4 carriers compared with non-ε4 carriers. Notes: x,y,z, coordinates of primary peak locations in the Talairach space. Abbreviation: DLPFC, dorsolateral prefrontal cortex; AI, anterior insula; BA, Brodmann area; L/R, left/right; SFG, superior frontal gyrus; aTP, anterior temporal pole; pMTG, posterior middle temporal gyrus; PCC, posterior cingulate cortex; vmPFC, ventromedial prefrontal cortex; IPC, inferior parietal cortex; AG, angular gyrus. (DOC) [file pone.0055902.s008.doc]

**Table S5.**

| Brain region | Side | BA | Cluster  Size  (mm^3^) | Talairach coordinates | | | Z Score |
| --- | --- | --- | --- | --- | --- | --- | --- |
|  |  |  |  | x | y | z |  |
| **Left DLPFC Network** | | | | | | | |
| SFG | L | 6/8 | 9976 | -15 | 33 | 52 | -3.47 |
| aTP | L | 38 | 6488 | -37 | 15 | -22 | -4.19 |
| pMTG | R | 39 | 5168 | 59 | -59 | 24 | -3.32 |
| aTP | R | 20 | 4848 | 47 | -3 | -24 | -3.80 |
| SFG | R | 6/8 | 4328 | 11 | 25 | 52 | -3.29 |
| **Left AI Network** | | | | | | | |
| PCC | L/R | 31 | 9784 | -5 | -59 | 26 | -3.69 |
| DLPFC | R | 9 | 7768 | 47 | 27 | 34 | -3.59 |
| Cuneus | R | 19 | 4720 | 51 | -77 | -6 | -4.04 |
| vmPFC | L | 11 | 4696 | 17 | 55 | -18 | -3.36 |
| IPC/AG/pMTG | L | 40/39 | 4344 | -45 | -59 | 30 | -3.57 |
